# Explaining sex differences in risk of bloodstream infections using mediation analysis in the population-based HUNT study in Norway

**DOI:** 10.1038/s41598-022-12569-8

**Published:** 2022-05-19

**Authors:** Randi Marie Mohus, Lise T. Gustad, Anne-Sofie Furberg, Martine Kjølberg Moen, Kristin Vardheim Liyanarachi, Åsa Askim, Signe E. Åsberg, Andrew T. DeWan, Tormod Rogne, Gunnar Skov Simonsen, Tom Ivar Lund Nilsen, Bjørn Olav Åsvold, Jan Kristian Damås, Erik Solligård

**Affiliations:** 1 Gemini Center for Sepsis Research, Institute of Circulation and Medical Imaging, NTNU, Norwegian University of Science and Technology, Trondheim, Norway; 2 Clinic of Anesthesia and Intensive Care, St. Olavs Hospital, Trondheim University Hospital, Torgarden, Postboks 3250, 7006 Trondheim, Norway; 3 Nord-Trøndelag Hospital Trust, Levanger, Norway; 4 Faculty of Health Sciences, Nord University, Levanger, Norway; 5 Department of Microbiology and Infection Control, University Hospital of North Norway, Tromsø, Norway; 6 Faculty of Health and Social Sciences, Molde University College, Molde, Norway; 7 Department of Infectious Diseases, St. Olavs Hospital, Trondheim University Hospital, Trondheim, Norway; 8 Department of Chronic Disease Epidemiology and Center for Perinatal, Pediatric and Environmental Epidemiology, Yale School of Public Health, New Haven, CT USA; 9 Research Group for Host-Microbe Interaction, Faculty of Health Sciences, UiT – The Arctic University of Norway, Tromsø, Norway; 10 Norwegian Institute of Public Health, Oslo, Norway; 11 Department of Public Health and Nursing, NTNU, Norwegian University of Science and Technology, Trondheim, Norway; 12 Department of Public Health and Nursing, K.G. Jebsen Center for Genetic Epidemiology, NTNU, Norwegian University of Science and Technology, Trondheim, Norway; 13 Department of Endocrinology, Clinic of Medicine, St. Olavs Hospital, Trondheim University Hospital, Trondheim, Norway; 14 Department of Clinical and Molecular Medicine, Centre of Molecular Inflammation Research, NTNU, Norwegian University of Science and Technology, Trondheim, Norway

**Keywords:** Infectious diseases, Immunology, Microbiology

## Abstract

Previous studies indicate sex differences in incidence and severity of bloodstream infections (BSI). We examined the effect of sex on risk of BSI, BSI mortality, and BSI caused by the most common infecting bacteria. Using causal mediation analyses, we assessed if this effect is mediated by health behaviours (smoking, alcohol consumption), education, cardiovascular risk factors (systolic blood pressure, non-HDL cholesterol, body mass index) and selected comorbidities. This prospective study included 64,040 participants (46.8% men) in the population-based HUNT2 Survey (1995–1997) linked with hospital records in incident BSI. During median follow-up of 15.2 years, 1840 (2.9%) participants (51.3% men) experienced a BSI and 396 (0.6%) died (56.6% men). Men had 41% higher risk of first-time BSI (95% confidence interval (CI), 28–54%) than women. Together, health behaviours, education, cardiovascular risk factors and comorbidities mediated 34% of the excess risk of BSI observed in men. The HR of BSI mortality was 1.87 (95% CI 1.53–2.28), for BSI due to *S. aureus* 2.09 (1.28–2.54), *S. pneumoniae* 1.36 (1.05–1.76), *E. coli* 0.97 (0.84–1.13) in men vs women. This study shows that men have higher risk of BSI and BSI mortality than women. One-third of this effect was mediated by potential modifiable risk factors for incident BSI.

## Introduction

Bloodstream infection (BSI) is a major global burden and may lead to sepsis which constitutes up to 60% of the global mortality burden^1,2^. The risk of acquiring BSI depends on the bacterial virulence, host characteristics, geographical location, and biological factors^1,3–8^. Epidemiological studies indicate a male predominance in BSI and sepsis. Nevertheless former studies on sex differences in incidence and mortality of BSI and sepsis have given conflicting results with increased risk in women^1^, increased risk in men^6,9^, increased mortality in women^10^, or increased mortality in men^1,11,12^. Importantly, disparities in immune function between sexes may arise from differences in biological characteristics such as anatomy and hormonal status, medical conditions, health behaviours, lifestyle, and exposure to different pathogens^13,14^. Most previous studies on sex differences in BSI and sepsis have been performed in small and selected cohorts, mainly from the intensive care unit (ICU)^6,10,11^, and there are limited population-based studies^9,15,16^ which better account for selection bias^17^. Studies on severe infections and sepsis tend to *adjust* for sex in their analyses^18^ but the mechanisms behind the observed sex differences are unexplored^19^. Little is known whether conditions that are known to increase BSI risk, like health behaviours^4^ cardiovascular disease risk factors or comorbidity^20,21^, contribute to the observed difference in risk of BSI between men and women. Such knowledge may help identify targets for intervention to reduce BSI and sepsis risk.

To assess the impact of sex as a risk factor for first-time BSI, BSI mortality, and BSI caused by the most common infecting bacteria, *Staphylococcus (S.) aureus, Streptococcus (S.) pneumoniae* and *Escherichia (E.) coli* we used data from the Norwegian HUNT study linked with prospectively recorded BSI episodes. Further, we examined if sex differences in health behaviours and education attainment, cardiovascular risk factors and selected comorbidities, which reflect known risk factors for BSI^4,20,21^ may explain the observed sex difference in risk of first-time BSI. We applied sequential mediation analysis^22^ using inverse-odds weighting^23^ to explore their potential mediating effect on the associations between sex and BSI.

## Methods

### Study population

The HUNT Study is a population-based health study conducted in the Nord-Trøndelag region in Norway and consists of four consecutive surveys inviting the total adult population approximately every 10th year. The second survey (HUNT2, 1995–1997), invited all adult inhabitants ≥ 20 years (*n* = 93,898) to a clinical examination and a comprehensive self-report of health-related topics. Of these, 65,237 (69%) chose to participate. The HUNT study database is regularly updated with information on date of migration and death from the National Registry. More details on the HUNT study are published elsewhere^24^. For the purpose of the present study, we excluded 47 (0.07%) participants who had a prior positive blood culture and 1150 (1.8%) who migrated or died before start of follow-up. A total of 64,040 participants were eligible for analyses (Supplemental Fig. S1).

### Measures

The exposure is sex as registered in the National Registry. The two main outcomes were first-time BSI and BSI mortality. The participants were followed for incident BSI by linkage to the Nord-Trøndelag Hospital Trust (HNT HF) Sepsis Registry using the personal identification number of Norwegian citizens^25^. All BSIs were confirmed at the microbiology laboratories at Levanger Hospital which provided all microbiology services in the Nord-Trøndelag region or at St. Olavs Hospital. Details about HNT HF sepsis registry are included in the supplemental material. We defined BSI mortality as death occurring within 30 days after detection of any BSI. In secondary analyses we assessed first**-**time BSI caused by the most common bacteria *E. coli*, *S. aureus* and *S. pneumonia,* and performed age-stratified analyses*.*

**Figure 1:**
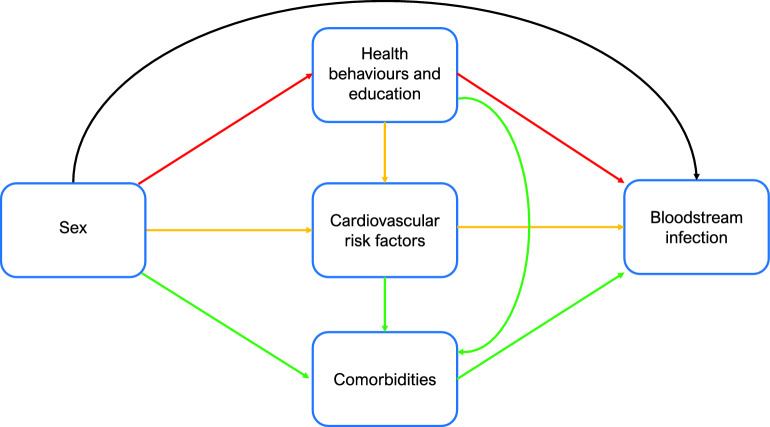
Mediation analysis. Diagram of the direct and indirect (i.e., mediated) effects of *sex* on *bloodstream infection*. The black arrow represents the natural direct effect of the association. Red arrows represent model 1, proportion mediated by health behaviours and education attainment. Yellow arrows represent model 2, proportion mediated jointly by health behaviours, education, and cardiovascular risk factors. Green arrows represent model 3, proportion mediated jointly by health behaviours, education, cardiovascular risk factors and comorbidities. Model 1) Smoking, alcohol use and educational attainment. Model 2) Systolic blood pressure, non-high-density lipoprotein cholesterol and Body Mass Index. Model 3) Cardiovascular disease, chronic kidney disease, diabetes, history of cancer, and chronic lung disease.

Mediators are variables that are causally located between exposure and outcome variables, and that partly explain the effect of the exposure on outcome^22,26^. Mediation analysis can assess indirect and direct effects and estimate the proportion of the total effect that works through the mediator of interest (i.e. proportion mediated)^27^. We used three distinct sets of mediators measured at inclusion to HUNT2; (1) health behaviours (smoking and alcohol use) and educational attainment; (2) cardiovascular risk factors (body mass index (BMI, kg/m^2^), systolic blood pressure (mmHg) and non-high-density lipoprotein cholesterol (non-HDL cholesterol, mmol/L); (3) comorbidities defined by self-report of cardiovascular disease (history of myocardial infarction, angina pectoris, and/or stroke), diabetes, cancer history, lung disease (asthma or chronic obstructive pulmonary disease) and standardised measurements of kidney function (estimated glomerular filtration rate (eGFR) < 60 mL/min/1.73 m^2^). The three sets of mediators reflect known risk factors for BSI^4,20,21^. For some of the included mediators there are reports of sex differences in prevalence, pathophysiology and outcomes^28,29^. The proposed diagram for the relationship between sex and risk of BSI is shown in Fig. 1. The aim of the analysis was to examine to which extent sex differences in risk of BSI may be related to these mediating factors. Details about the measurements and categorisation of mediators are included in the Supplemental Material.

### Statistical analyses

We used Cox proportional hazard regression to estimate the hazard ratios (HRs) with 95% confidence intervals (95% CI) of a first-time BSI and of BSI mortality in men compared to women. Attained age was used as the time scale. Start of follow-up was defined by the availability of data in the sepsis registry. For patients referred to St. Olavs hospital, the tertiary referral centre, BSI information was included depending on their primary hospital. Participants contributed person-years from inclusion date in HUNT2 except for participants having Namsos as their primary hospital, they contributed from 1 September 1999.

In the analysis of BSI risk, participants were followed until their first BSI. For BSI-mortality participants were followed until death within 30 days of any BSI episode. For both analyses participants were censored at time of migration out of Nord-Trøndelag, death of all causes, or end of follow-up set to 31 December 2011, whichever occurred first. The proportional hazards assumption was examined by visual inspection of log–log plots and tests of Schoenfeld residuals. Using Stata *stcompadj,* we estimated cumulative incidence and mortality from start of follow-up to first-time BSI and BSI mortality, accounting for death by all causes as a competing risk and we provide cumulative incidence and cumulative mortality curves to illustrate changes during follow-up. As supplemental analyses we assessed subhazard ratios taking death as a competing event into account for first-time BSI and BSI mortality.

In secondary analyses we estimated hazard ratios and cumulative incidence of first-time BSI caused by the most common infecting bacteria*.* Further, we conducted age-stratified analyses on risk of first-time BSI; < 50, 50 to < 65, 65 to 79, and ≥ 80 years to address whether menopause affects women’s risk of BSI, and to assess sex differences in BSI risk with advancing age. To examine the associations between the mediators included in Model 2 and risk of first-time BSI, we conducted sensitivity analyses using Cox regression for BMI, systolic blood pressure and non-HDL cholesterol.

For the mediation analysis we used an *inverse odds weighting (IOW)* procedure^23^. IOW is a counterfactual method that enables a decomposition of the *total effect* of the exposure (sex) on the outcome (first-time BSI) into a *natural direct effect (NDE)* from exposure on outcome, and a *natural indirect effect (NIE)* through multiple mediators^22,23^. The method accommodates multiple mediators simultaneously and is robust to unmeasured common causes of the mediators^22^.

The inverse odds weights were obtained by regressing the exposure on all mediators of interest with age as a covariate. In our analysis, the total effect is interpreted as the total association between sex and first-time BSI, the NIE is the proportion of excess BSI risk in men mediated by the risk factors, whereas the NDE is the proportion of excess BSI risk in men not associated with these factors. The proportion mediated is the percent of the total association that is mediated through the risk factors. We did not estimate the NIE of individual mediators separately as it may not be appropriate when the mediators affect each other or when single mediator-outcome confounders may be affected by exposure^22,30^. Instead, we estimated the NIE with a sequential approach using three models. In model 1, we assessed education attainment and health behaviours (smoking and alcohol use); in model 2, we added the cardiovascular risk factors (BMI, systolic blood pressure and non-HDL cholesterol) to address potential preclinical disease; and in model 3, the selected comorbidities (cardiovascular, diabetes, cancer history, lung, and kidney disease) were included to the complete set of mediators. This approach assumes that the cardiovascular risk factors and further the comorbidities are causal descendants of the health behaviours and educational attainment. The sequential approach further implies that model 3 reflects the best interpretation of the mediation analyses as all mediators and age are included^22^.

We performed bootstrapping based on 1000 replications to derive percentile-based CIs for all mediation parameters^31^, and the NDE and NIE are presented as HRs with 95% CIs. The proportion mediated on the log scale was calculated using the formula (lnHR_NIE_ /lnHR_TOTAL_)^23^. All statistical analyses were performed using Stata version 17.0. A detailed description of the IOW analyses is included in Supplemental Table S1.

### Ethical approval

The study was approved by the Regional Committee for Medical and Health Research Ethics of Central Norway (REK no 2012/153 and REK no 94135), and by the HUNT data access committee. Participation in HUNT 2 was voluntary, and informed written consent to data collection and linking their data to other registers was obtained from all participants. All methods were performed in accordance with the Declaration of Helsinki.

## Results

### Population characteristics

During a median follow-up of 15.2 years (IQR 12.3–15.5 years), among 64,040 participants (46.8% men), 1840 (2.9%) experienced a BSI and 396 (0.6%) died within 30 days after a BSI episode. The median age at inclusion was similar for both sexes. Both men and women who experienced a BSI were older (median age at inclusion 67.4 and 68.0 respectively), and they had a higher comorbidity burden than participants who did not have a BSI during follow-up (Table 1).

**Table 1:** Baseline characteristics of the study population at inclusion in HUNT2, n = 64,040.

	Men	Women
Total population n (%)	29,962 (46.8)	34,087 (53.2)
First-time BSI n (%)^1^	943 (51.3)	897 (48.7)
BSI mortality n (%)^2^	224 (56.6)	172 (43.4)
Age (mean, IQR)	48.6 (36.5–62.9)	48.7 (36.2–64.2)
**Smoking**		
Current (%)	8334 (27.8)	9726 (28.5)
Prior (%)	9422 (31.4)	6516 (19.1)
Never (%)	10,668 (35.6)	15,230 (44.7)
**Alcohol use**		
< 1 unit/2 weeks (%)	8448 (28.2)	16,069 (47.3)
1–7 units/2 weeks (%)	14,258 (47.6)	14,484 (42.6)
8–14 units/2 weeks (%)	4602 (15.4)	1861 (5.5)
≥ 15 units/2 weeks (%)	1643 (5.5)	272 (0.8)
**Education**		
< 10 years (%)	20,625 (68.9)	22,259 (65.3)
10–12 years (%)	2302 (7.7)	3436 (10.1)
> 12 years (%)	5650 (18.9)	6412 (18.8)
**BMI (kg/m**^**2**^**)**		
< 18.5 (%)	118 (3.9)	349 (1.0)
18.5–24.9 (%)	10,498 (35.0)	14,736 (43.2)
25–29.9 (%)	14,757 (49.3)	12,345 (36.2)
30–34.9 (%)	3674 (12.3)	4640 (13.6)
35–39.9 (%)	496 (1.7)	1236 (3.6)
≥ 40 (%)	74 (0.3)	344 (1.0)
Systolic blood pressure (mmHg) median (IQR)	137 (127–150)	131 (118–149)
Non-HDL cholesterol (mmol/L) median (IQR)	4.5 (3.7–5.3)	4.3 (3.5–5.3)
**Comorbidities**		
Cardiovascular disease^3^ (%)	2918 (9.7)	2014 (5.9)
Chronic kidney disease (%)	979 (3.3)	1802 (5.3)
Diabetes (%)	895 (3.1)	970 (2.9)
Cancer history (%)	878 (2.8)	1413 (4.1)
Chronic lung disease^4^ (%)	1183 (4.0)	1011 (3.0)

*BSI* bloodstream infection, *n* numbers, *IQR* interquartile range, *BMI* body mass index, *HDL* high-density lipoprotein.

^1^Percentage of total first-time BSI in both sexes.

^2^BSI mortality was defined as all-cause mortality within 30 days after a BSI. Percentage of BSI mortality on both sexes.

^3^History of myocardial infarction, angina pectoris and/or stroke.

^4^History of chronic obstructive pulmonary disease or asthma.

### Risk of BSI and BSI mortality

Men were more likely to experience a first-time BSI and to die from a BSI compared to women. Men had 1.41 (HR, 95% CI 1.28–1.54) times the risk of first-time BSI, and had 1.87 (HR, 95% CI 1.53–2.28) times the risk of dying from a BSI (Table 2) compared with women. In analyses by the most common infecting bacteria, men had a 2.09-fold (HR, 95% CI 1.28–2.54) risk of BSI caused by *S. aureus*, and 1.36 (HR, 95% CI 1.05–1.76) increased risk of *S. pneumonia*. The corresponding result for *E. coli* did not show higher risk in men with HR of 0.97 (95% CI 0.84–1.13) (Table 3).

**Table 2:** Associations of sex with risk of bloodstream infection and BSI mortality.

	Risk of first-time BSI adjusted for age^1^				BSI mortality^2^ adjusted for age^1^			
	Years at risk	No. BSI	HR	95% CI	Years at risk	No. BSI deaths	HR	95% CI
Women	436,758	897	1.0	Reference	472,012	172	1.0	Reference
Men	373,915	943	1.41	1.28–1.54	404,723	224	1.87	1.53–2.28

*BSI* bloodstream infection, *HRs* hazard ratios, *95% CI* 95% confidence intervals, *No.* numbers.

^1^Cox regression analyses were adjusted with age as the underlying scale.

^2^BSI mortality was defined as all-cause mortality within 30 days after a bloodstream infection.

**Table 3:** Associations of sex with risk of bloodstream infections caused by the most common bacteria.

	Years at risk	Risk of *S. aureus* BSI^1^			Risk of *S. pneumoniae* BSI^1^			Risk of *E. coli* BSI^1^		
		No. BSI	HR	95% CI	No. BSI	HR	95% CI	No. BSI	HR	95% CI
Women	436,758	83	1.0	Reference	113	1.0	Reference	399	1.0	Reference
Men	373,915	129	2.09	1.28–2.54	119	1.36	1.05–1.76	285	0.97	0.84–1.13

*BSI* bloodstream infection, *HRs* hazard ratios, *95% CI* 95% confidence intervals.

^1^Cox regression analyses were adjusted for age as the underlying scale.

**Figure 2:**
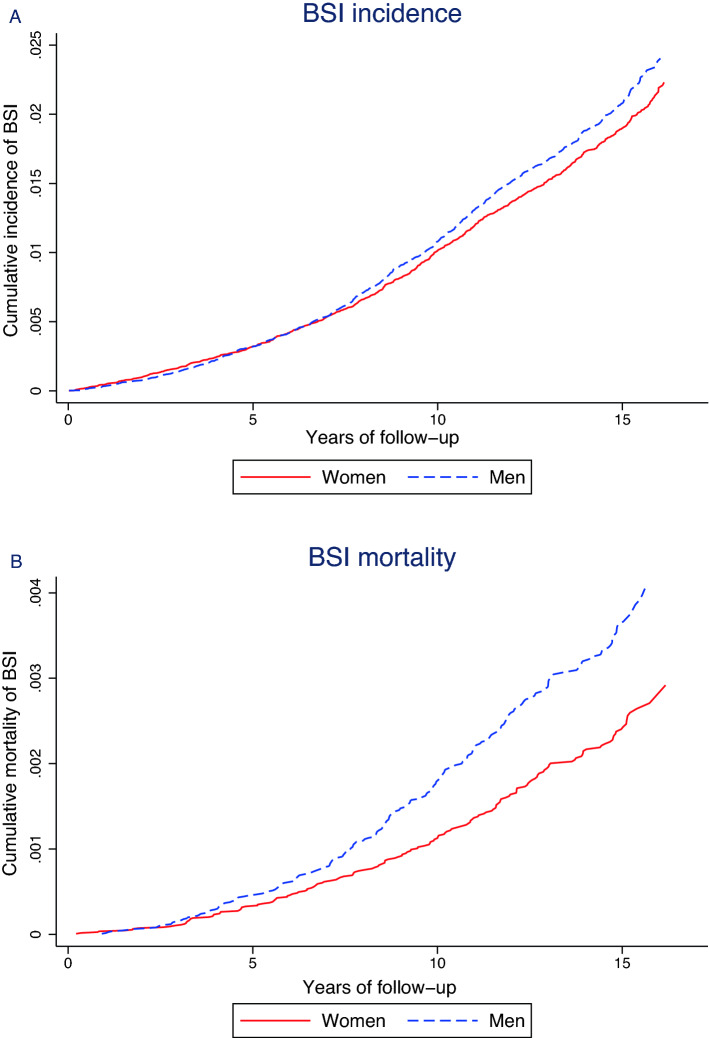
Sex differences in cumulative incidence and mortality of BSI. Age-adjusted sex difference in cumulative incidence of BSI (**A**), and in cumulative mortality (**B**), estimated for age 49.99 (the mean age of the total population). Note: due to the variation in incidence of different outcomes the scale of the Y-axis is not uniform across the panels.

**Figure 3:**
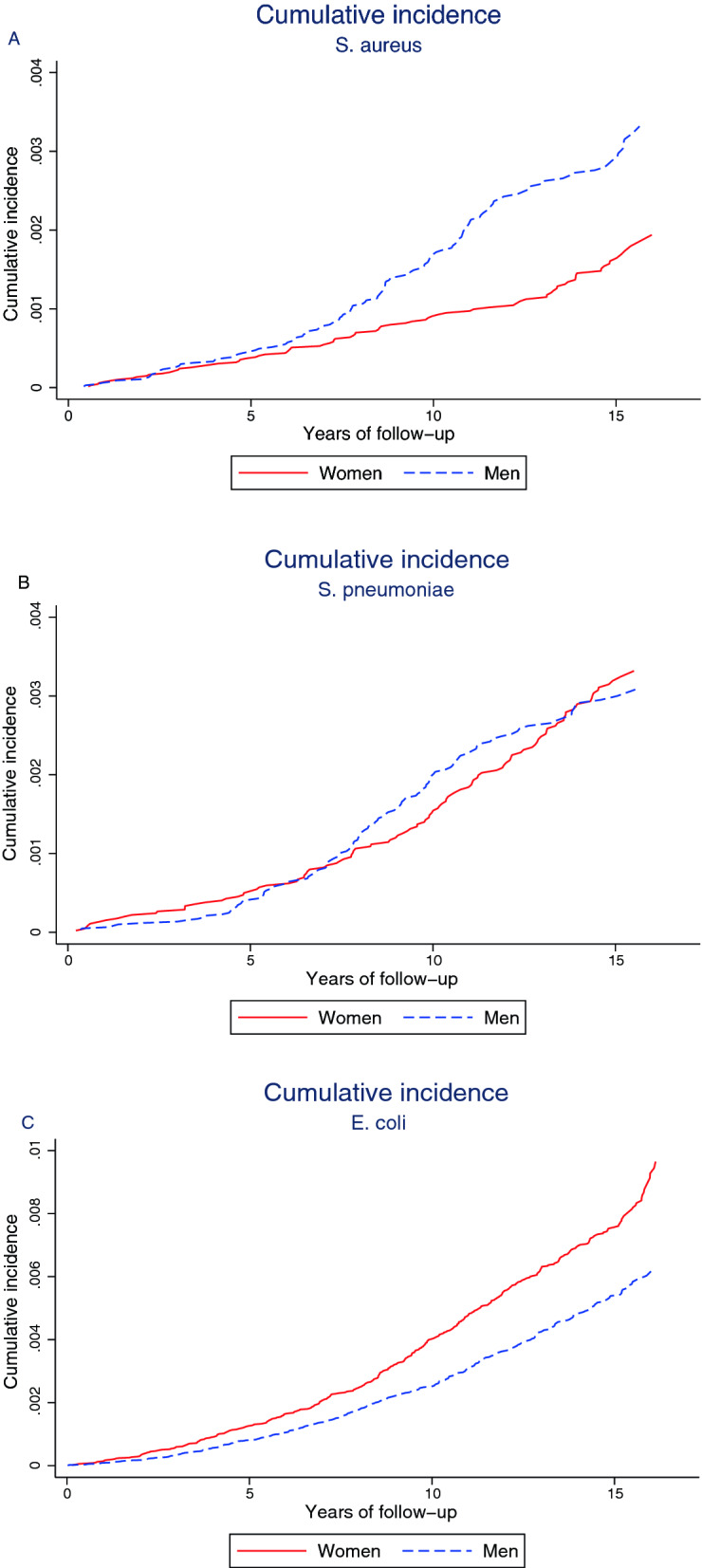
Sex differences in cumulative incidence of BSI caused by the most common bacteria. Age-adjusted sex difference in cumulative incidence of* S. aureus* (**A**),* S. pneumoniae* (**B**), and* E. coli* (**C**), estimated for age 49.99 (the mean age of the total population). Note: due to the variation in incidence of different bacteria the scale of the Y-axis is not uniform across the panels.

The above findings are illustrated by graphing the age-adjusted cumulative incidence of first-time BSI and cumulative BSI mortality in Fig. 2. The cumulative incidence of BSI was higher among men than women after the first five years of follow-up. For BSI mortality the sex difference was apparent after the first 2.5 years of follow-up and during follow-up the sex differences in mortality increased. The subhazards obtained for first-time BSI and BSI mortality, provide the same direction of the associations as the Cox regression analyses (Supplemental Table S2). We additionally present cumulative incidence curves for the three most common infecting bacteria in Fig. 3A–C. Men had higher cumulative incidence of *S. aureus*, especially after the first seven years of follow-up, whereas *E. coli* had higher cumulative incidence among women.

The age-stratified analyses at estimated menopause (< 50 years) did not reveal any clear sex difference in risk of BSI before menopause. However, the youngest age-group had few episodes of BSI. In the older age-groups we observed an increased incidence of BSI in both men and women, where men show a substantially increased risk of BSI as they age compared to women (Supplemental Table S3).

### Mediation analyses

In Table 4 we present the total effect, the natural direct and indirect effects of sex on risk of first-time BSI. Compared with women men had an estimated HR of 1.40 (95% CI 1.24–1.55) for first-time BSI. Behavioural risk factors and education mediated 10% (model 1), after adding the cardiovascular risk factors the proportion mediated was reduced to 5% (model 2), whereas the whole set of mediators, including comorbidities, jointly mediated 34% of the total effect (model 3).

**Table 4:** Mediation of the associations between sex and BSI by behavioural risk factors, educational attainment, cardiovascular risk factors and comorbidities.

Mediation by behavioural risk factors^1^ and education	Risk of first-time BSI
	HRs (95% CI^a^)^b^
**Model 1**	
Total effect	1.40 (1.24–1.55)
Natural direct effect (NDE)	1.36 (1.18–1.57)
Natural indirect effect (NIE)	1.04 (0.97–1.07)
Proportion mediated^c^	10%

Mediation by behavioural risk factors^1^, education, and cardiovascular risk factors^2^	HRs (95% CI^a^)^b^
**Model 2**	
Total effect	1.40 (1.24–1.55)
Natural direct effect (NDE)	1.38 (1.19–1.58)
Natural indirect effect (NIE)	1.02 (0.92–1.07)
Proportion mediated^c^	5%

Mediation by behavioural risk factors^1^, education, cardiovascular risk factors^2^ and comorbidity risk factors^3^	HRs (95% CI^a^)^b^
**Model 3**	
Total effect	1.40 (1.24–1.55)
Natural direct effect (NDE)	1.25 (1.05–1.47)
Natural indirect effect (NIE)	1.12 (1.02–1.17)
Proportion mediated^c^	34%

*BSI* bloodstream infection, *HRs* hazard ratios, *95% CI* 95% confidence intervals.

^1^Smoking, alcohol use and educational attainment at baseline.

^2^Systolic blood pressure, non-high-density lipoprotein cholesterol and Body Mass Index.

^3^Cardiovascular disease, chronic kidney disease, diabetes, history of cancer, or chronic lung disease.

^a^Percentile-based bootstrap CIs are reported.

^b^Estimates are adjusted for age as a covariate.

^c^Proportion mediated: (ln HR_NIE_/ln HR_TOTAL_).

To examine the reduction in proportion mediated from 10 to 5% in model 2, in sensitivity analyses, we observed an increased risk of BSI in persons with low BMI (< 18.5) and in persons with increasing BMI compared to the normal BMI group (18.5–24.4). For systolic blood pressure we did not observe any risk difference, and for non-HDL cholesterol the HR suggested a protective effect but with imprecise estimates (Supplemental Table S4).

## Discussion

In this large Norwegian population-based study with a follow-up of more than 15 years, male sex was associated with 41% higher risk of BSI and 87% higher risk of dying from a BSI. An estimated 34% of the increased risk of BSI in men was mediated by known BSI risk factors. We additionally found that men had 2.09 times the risk of BSI caused by *S. aureus* compared to women*.* These findings add weight to the observed male preponderance seen in severe infections and point out modifiable BSI risk factors that are targets for preventive measures to reduce the burden of BSI.

There are few population-based studies on sex differences in the epidemiology of BSI and to our knowledge, no previous studies have performed mediation analysis to explain the sex differences of BSI. We used the IOW method which is known to be robust using multiple mediators en bloc and the rich baseline information from HUNT2 allowed us to implement mediation analyses in a time-to-event context^22,23^. The sequential approach enabled us to examine if the observed excess risk in men was mediated through different known risk factors for BSI. For many medical conditions men and women differ regarding incidence, the underlying pathophysiology and responses to therapy^28,29^. For health behaviours, more men reported smoking, and they reported higher alcohol use. We also observed higher prevalence of obesity among women in HUNT2. Adding cardiovascular risk factors to health behaviours and education, the proportion mediated lowered from 10 to 5%, which indicates some interactions or common pathways for these mediators^22^. This result might be due to some of the mediators included in model 2 being more frequent or harmful in women, or the mediators might reduce the risk of BSI. The complete model with all mediators included accommodates the assumptions required, and most likely reflects the best modelling of the associations^22,30^ explaining 34% of the excess BSI risk in men. Interventions to reduce modifiable risk factors in the population will likely reduce the burden of BSI, particularly in older men with high burden of known BSI risk factors.

The population-based design ensures that all BSI occurring in residents of a defined geographical area are included, which is an advantage over ICU cohorts^17^. Our results are supported by one study including 1051 patients, showing that men had higher risk of BSI. Like our study they described that BSI incidence increases by age, and men had twice the rate of *S. aureus* BSI^15^. Another population-based study comprising 9266 patients with BSI admitted to ICU found that male sex is a risk factor for BSI^9^. A recent study restricted to persons aged ≥ 65, found that men were at increased risk of BSI compared to females (incidence rate ratio 1.44, 95% CI 1.32–1.59) and the sex difference was most pronounced in the oldest patients, similar to our results^16^. On the other hand, the Global Burden of Disease Study found that age-standardised sepsis incidence was higher among women, while sepsis-related mortality was higher among men^1^. This study included results from 195 countries and comprised all age groups. They found higher sepsis incidence in low-income countries, and the pattern of sepsis incidence and mortality varied according to location, which is not directly comparable to our study population.

We identified higher BSI mortality in men which is in line with a recent study of infection related death in UK Biobank^12^. Conversely, some ICU studies report higher sepsis-related mortality in women^10,29^. A recent meta-analysis evaluating the associations between sex and mortality in critically ill adults showed inconclusive results^30^. This conflicting evidence concerning sex differences in mortality is most likely due to the heterogeneity of BSI and sepsis depending on the aetiology and the cohort studied^17^, but may also be affected by sex differences in immune responses^13,14,32^ and differences in treatment^10,18^.

The second most common infecting agent in our study was *S. aureus* which was far more common in men. *S. aureus* is associated with superficial infections of soft tissues with the potential for invasive infections and is the most important cause of BSI-associated death^33^. Previous studies indicate higher probability of nasal colonization in men, which is a risk factor for invasive *S. aureus* infections^34^. Other studies show that testosterone levels and use of hormone contraceptives among females alter nasal colonization, indicating that sex hormones affect the immune response to *S. aureus*^35,36^. The higher prevalence of *S. aureus* colonization in men is of particular interest, as preventive measures like eradication or temporary suppression could lower the risk of invasive infections which is especially important in hospitalized patients^37^.

In a sensitivity analysis we found that the sex differences in BSI risk are evident after predicted age of menopause indicating that alterations in both innate and adaptive immune functions with age may be sex specific. Aging is associated with chronic inflammation and a generally reduced immune function. Sex hormone levels in men and women change with age. Women face an abrupt decline during menopause, whereas men have a steady decline from second decade of life^38^. As in former studies, our study points out that advancing age is a risk factor for developing and dying from BSI and elderly men are at particular risk^9,15,16^.

Major strengths of our study include its large size, the population-based design, long-term follow-up and linkage to microbiological records which represent the gold standard to identify BSI within a population^17^. Our definition of BSI as a laboratory verified positive blood culture, excluding blood cultures solely with microorganisms associated with skin contamination, ascertains the accuracy of the outcome studied. In addition, reviews of medical records of patients with *S. aureus* and *S. pneumoniae* BSI in this cohort showed that ~ 98% met the 2001 sepsis criteria^32,39^. We were able to study BSI incidence and BSI mortality in a large population without the potential referral bias seen in single institution studies of BSI. The complete ascertainment of all BSI, together with the rich baseline measurements of known BSI risk factors in HUNT2, allows for an accurate estimation of incidence and mortality in the population, with the potential of risk factor identification and mediation analyses.

There are some limitations of our study that merit attention. First, we lack information on immunosuppressive medication which are known risk factors for BSI and BSI mortality^40^. We did not have information on the clinical course after detection of a BSI. Therefore, we did not perform mediation analyses on risk of BSI mortality as in-hospital factors such as correct and timely antibiotics and resuscitation measures strongly influence mortality^41^. Second, investigating BSI is dependent on clinician’s suspicion and decision to submit blood cultures for testing, with the chance of some undetected cases. Further, we cannot rule out if the clinical presentation of infections is different in men and women, and that this could result in disproportionate blood culture sampling depending on sex. Another concern would be if the clinical presentation of infection led to disproportionate and sex dependent hospital admissions. Third, the mediators were only measured once at inclusion to HUNT2 and could have changed during the 16 years of follow-up. This potential misclassification would most likely lead to underestimation of the mediating effect. Forth, the subjective assessment of some mediators, and dichotomised mediators, are more prone to possible mediator misclassification. This could lead to underestimation of the indirect effect and overestimation of the direct effect^22^. Fifth, we were not able to assess the mediators individually, as this could have violated the model assumptions^22,30^.

Despite these limitations, our study provides a foundational observation of the existing sex differences in BSI epidemiology and adds important information for clinicians, researchers and policymakers concerning BSIs. Our results suggest that sex disparities in BSI cannot be explained fully by the mediating factors investigated. Sex affects the shape of immune responses attributed to genetic, hormonal, and environmental factors^13,14,32^. The human X-chromosome encodes a number of critical genes involved in the regulation of immune functions^32^. It is clear that sex extensively influences the host immune responses, but this sexual dimorphism is underappreciated, and sex bias is a major challenge in clinical trials^18^. Sex hormones act as important modulators of immune functions and responses; testosterone and progesterone are immunosuppressive, while oestradiol is immunoenhancing^14^. Few human studies have investigated sex hormones’ effects in severe infections and sepsis. Interestingly, in covid-19 where men are more prone to a severe course, the use of antiandrogens in men have shown promising results on severity^42^. Furthermore, in a review of health records in post-menopausal women with regular use of oestradiol, the fatality risk of covid-19 is reduced by more than 50%^43^.

Future perspectives of our results include the need for targeted research on how these sex differences could be addressed to achieve a longer and healthier life for both men and women. Additional work should focus on how health behaviours, education level, cardiovascular risk factors and comorbidities play a role in the sex disparities seen in severe infectious diseases. Knowledge of mediating factors together with recognition of sex differences in severe infections are important for public health leaders, researchers, and clinicians as it can inform preventive actions and identify individuals especially at risk^19^.

## Conclusion

Our study has shown that men have an increased risk of BSI and BSI mortality. Using mediation analyses we estimated that 34% of the increased risk of BSI is mediated through known BSI risk factors. As BSI represents an important global burden of disease, our study serves as a catalyst for additional investigations by establishing the presence of sex differences and mediating risk factors. This will potentially lead to targeted management strategies to prevent BSI and sepsis in both men and women.

## Supplementary Information

Supplementary Information.

## Data Availability

Data is available from the authors upon reasonable request and by application to HUNT Research Centre. https://hunt-db.medisin.ntnu.no/hunt-db/.

## Code availability

A detailed description of the IOW analyses is included in Supplemental Material.
