# Supplementary material for: Explaining sex differences in risk of bloodstream infections using mediation analysis in the population-based HUNT study in Norway
Source: Sci Rep. 2022 May 19;12:8436. doi: 10.1038/s41598-022-12569-8 (PMC9118181; doi:10.1038/s41598-022-12569-8)
Supplement: Supplementary file 1 — Supplementary Information. [file 41598_2022_12569_MOESM1_ESM.docx]

### **Supplementary Information**

# Explaining sex differences in risk of bloodstream infections using mediation analysis in the population-based HUNT study in Norway

Randi Marie Mohus

Lise T. Gustad

Anne-Sofie Furberg

Martine Kjølberg Moen

Kristin Vardheim Liyanarachi

Åsa Askim

Signe E. Åsberg,

Andrew T. DeWan

Tormod Rogne

Gunnar Skov Simonsen

Tom Ivar Lund Nilsen

Bjørn Olav Åsvold

Jan Kristian Damås

Erik Solligård

**Supplemental to methods:** Study population and The Nord-Trøndelag Hospital Trust Sepsis Registry

Measurements and categorization of mediators.

**Supplemental Figure S1:** Flow Chart of Study Recruitment and Follow-up

**Supplemental Table S1:**  Procedure of estimating mediation parameters using the IOW method.

Supplemental Table S2: Age-stratified analyses of the associations of sex with risk of bloodstream infection

**Supplemental Table S3:** Subhazards of the association between sex and first-time BSI and BSI mortality defining death by other causes than BSI as a competing risk event

**Supplemental Table S4**: Association between BMI groups and risk of first-time BSI adjusted for age and sex

# Supplemental to methods

# Study population and The Nord-Trøndelag Hospital Trust Sepsis Registry

The HUNT study database is regularly updated with information on date of migration and death from the National Registry. The Nord-Trøndelag region in Norway has a population of 130,000, where approximately 70% of the population is served by Levanger hospital and 30% is served by Namsos hospital. The tertiary referral centre is St. Olavs hospital in Trondheim. The population is stable with a net out-migration of 0.3% per year, and ethnically homogeneous (97% Caucasians) ^1^.

The Nord-Trøndelag Hospital Trust (HNT HF) Sepsis Registry has prospectively recorded information on all clinically relevant BSI events at Levanger Hospital from 1 January 1995, and Namsos Hospital was included in the registry from 1 September 1999. The microbiology laboratory at Levanger Hospital exclusively provided all microbiology services for the two hospitals in Nord-Trøndelag region ^2^. In addition, all HUNT2 participants with a positive blood culture recorded at St. Olavs hospital were included in the registry from 1 January 1995 to assure completeness of the study cohort. For all analyses using first-time BSI or first-time BSI caused by *E. coli,* *S. aureus or S. pneumoniae* as the outcome the first registered date of a positive blood culture from either of the microbiology labs at Levanger or St. Olavs hospitals decided the episode. BSI mortality was based on information on every BSI event with information on dates of positive blood cultures from both microbiology labs. BSI mortality was defined as all cause death within 30 days after a BSI episode. Data from all hospitals were available through 2011. Blood cultures solely with microorganisms associated with skin contamination such as coagulase negative *Staphylococcus* species*, Corynebacterium* species and *Cutibacterium* species were not considered as BSI ^3^.

Mediators

Health behaviours were defined by smoking status and alcohol use. Smoking was defined from several questions on past and current smoking; as “current smoking” (smoking tobacco daily), “prior smoking” (any prior daily tobacco smoking) or “never smoked”. Alcohol use as “never drink alcohol”, “1−7 units of alcohol in two weeks”, “8−12 units alcohol in two weeks” or “more than 15 units in two weeks”.

Educational attainment was categorized as <10 years, 10−12 years and >12 years of schooling.

Cardiovascular risk factors were defined as body mass index (BMI), blood pressure, cholesterol and estimated glomerular filtration rate (eGFR). BMI was calculated as weight (kg) divided by the squared value of height (m^2^), measured by trained nurses at the clinical examination at inclusion in HUNT2 with the participants wearing light clothing and no shoes. BMI was categorized as recommended by WHO (<18.5, 18.5−24.9, 25−29.9, 30−34.9, 35−39.9, ≥ 40 kg/m^2^). Systolic blood pressure was measured three times at 1-minute intervals using an automatic oscillometric method (Dinamap, Critikon, Florida, USA) after a person had come to rest, with cuff size adjusted to arm circumference. We used the mean of the second and third blood pressure measurements. Serum total and high-density lipoprotein (HDL) cholesterol were analysed using enzymatic colorimetric methods (Boeheringer Mannheim, Germany). Non-HDL cholesterol was calculated as the difference between total and HDL cholesterol. The creatinine values used in the eGFR calculation were measured in non-fasting serum blood samples drawn by trained nurses and analyzed at the Central Laboratory at Levanger Hospital. eGFR was estimated from recalibrated creatinine values using the Modification of Diet in Renal Disease (MDRD)-formula ^4^.

Supplemental Figure S1: Flow Chart of Study Recruitment and Follow-up

Invited to HUNT2 (1995-97)

*n* = 93,898

Accepted invitation

*n* = 65,237

Censoring:

Positive blood culture prior to follow-up,

*n* = 47

Died or migrated prior to follow-up,

*n* = 1150

Included in follow-up^a^

*n* = 64,040

First-time episode of BSI

*n* = 1840

a) Follow-up for residents belonging to Levanger hospital and for patients referred to St. Olavs hospital (tertiary referral center): From entry date in HUNT2.

a) Follow-up for residents belonging to Namsos hospital: From 1 September 1999.

Death within 30 days after any episode of BSI

*n* = 396

Supplemental Table S1: Procedure of estimating mediation parameters using the IOW method* ^5,6^

| Steps | Procedure | Stata code |
| --- | --- | --- |
|  | Preparing the data | *User written program to estimate mediation parameters  Capture program drop IOW  Program IOW, rclass  Capture drop predprob inverseodds wt_iow |
| Step 1: Exposure model | The exposure model is run by regressing the exposure on all mediators and age as a covariate using logistic regression | *model 1  logit Sex i. smoking i. alc i. edu_cat age  *model 2  logit Sex i. smoking i. alc i. edu_cat systBP non_HDL i. BMI age  *model 3  logit Sex i. smoking i. alc i. edu_cat systBP non_HDL i. BMI i. LungDis i. CardDis i. diabetes i. cancer i. RenalDis age |
| Step 2: Create inverse odds weights | Based on the logistic regression models in step 1, the inverse odds weights are created by estimating the inverse of the predicted odds for each observation in the exposed group. The exposed and unexposed groups are then reweighted as follows: exposed = inverse odds, unexposed = 1 | *obtain predicted probability for each individual based on the above regression models:  predict predprob, p  *calculate each individual’s inverse odds from the predicted probability:  gen inverse odds = ((1-preprob)/predprob)  gen wt_iow = 1 if sex==0  replace wt_iow = inverseodds if sex==1 |
| Step 3: Total effect model | The total effect of the exposure is estimated by using Cox regression model | stset eof, id(PID) failure(bacteriemia) origin(birthyear) enter(Enterdate) scale(365.25)  stcox sex  matrix bb_total = e(b)  scalar b_total =bb_total [1,1]  return scalar b_total=bb_total [1,1] |
| Step 4: Natural direct effect model | The direct effect model is similar to the total effect model, but includes the inverse odds weight constructed from the mediators, instead of controlling for the mediators themselves. | *Estimate the direct effect of sex on BSI by means of a weighted Cox proportional hazards model with the weights (pweight=wt_iow) achieved in step 2:  stset eof [pweight=wt_iow], id(PID) failure(bacteriemia) origin(birthyear) enter(Enterdate) scale(365.25)  stcox sex  matrix bb_direct = e(b)  scalar b_direct = bb_direct [1,1]  return scalar b_direct = bb_direct [1,1] |
| Step 5: Natural indirect effect model | The indirect effect is estimated by subtracting the direct effect from the total effect. | return scalar b_indirect=b-total-b_direct |
| Step 6: Proportion mediated | The proportion mediated is estimated using the formula: lnHR_NIE_ /lnHR_TOTAL_ | return scalar b_mediated=((b_indirect)/b_total)  end |
| Step 7: Estimate standard errors | The standard errors and confidence intervals are estimated by bootstrapping. | bootstrap r(b_indirect) r(b_direct) r(b_total) r(b_mediated), seed (32222) reps(1000):IOW  estat bootstrap, all |

*) Stata 17.0 was used in all statistical analyses.

Supplemental Table S2: Age ^1^-stratified analyses of the associations of sex with risk of bloodstream infection

|  |  |  | Risk of first-time BSI | | |
| --- | --- | --- | --- | --- | --- |
| Age group | Sex | Years at risk | No of BSI | HR | 95% CI |
| < 50 years | Women | 243943 | 162 | 1.00 | Reference |
|  | Men | 215523 | 144 | 0.99 | 0.79 – 1.24 |
| ≥ 50 – < 65 years | Women | 107835 | 228 | 1.00 | Reference |
|  | Men | 97375 | 263 | 1.31 | 1.10 – 1.57 |
| ≥ 65 – < 80 years | Women | 74200 | 432 | 1.00 | Reference |
|  | Men | 55512 | 455 | 1.53 | 1.34 – 1.75 |
| ≥ 80 years | Women | 10778 | 75 | 1.00 | Reference |
|  | Men | 5503 | 81 | 2.17 | 1.58 – 2.97 |

BSI: Bloodstream infection HR = hazard ratio. 95% CI = 95% confidence intervals. No. = Numbers

1) Age at inclusion in HUNT2

**Supplemental Table S3: Subhazards^1^ of the association between sex and first-time BSI and BSI mortality defining death by other causes than BSI as a competing risk event**

|  | **Years at risk** | **No. competing events** | **Subhazard ratio** | **95% CI** |
| --- | --- | --- | --- | --- |
| First-time BSI | 810674 | 10805 | 1.20 | 1.10 – 1.32 |
| BSI mortality | 876744 | 10805 | 1.52 | 1.25 – 1.86 |

BSI: Bloodstream infection, No. = Numbers, 95% CI = 95% confidence intervals.

1. Using Stata command stcrreg.

**Supplemental Table S4: Association between BMI groups and risk of first-time BSI adjusted for age^1^ and sex^2^**

| **BMI** | **Years at risk** | **No. BSI** | **HR** | **95% CI** |
| --- | --- | --- | --- | --- |
| <18.5 | 5270 | 15 | 1.75 | 1.04 – 2.92 |
| 18.5 - 25 | 320605 | 523 | Reference |  |
| 25 – 29.9 | 348436 | 813 | 1.03 | 0.93 – 1.16 |
| 30 – 34.9 | 103977 | 339 | 1.31 | 1.14 - 1.50 |
| 35 – 39.0 | 21402 | 90 | 1.87 | 1.49 – 2.34 |
| ≥40 | 5188 | 33 | 3.09 | 2.17 - 4.41 |
| Systolic blood pressure^3^ | 802764 | 1805 | 1.00 | 0.999 – 1.001 |
| Non-HDL cholesterol^3^ | 803177 | 1809 | 0.97 | 0.93 – 1.01 |

1. Adjusted for age as underlying time-scale.
2. Stratification by sex.
3. Additionally adjusted for BMI.

# References

1. Krokstad, S.*, et al.* Cohort Profile: The HUNT Study, Norway. *Int J Epidemiol* **42**, 968-977 (2012).

2. Mehl, A.*, et al.* Burden of bloodstream infection in an area of Mid-Norway 2002-2013: a prospective population-based observational study. *BMC Infect Dis* **17**, 205 (2017).

3. Hall, K.K. & Lyman, J.A. Updated review of blood culture contamination. *Clin Microbiol Rev* **19**, 788-802 (2006).

4. Hallan, S., Astor, B. & Lydersen, S. Estimating glomerular filtration rate in the general population: the second Health Survey of Nord-Trondelag (HUNT II). *Nephrol Dial Transplant* **21**, 1525-1533 (2006).

5. Nguyen, Q.C., Osypuk, T.L., Schmidt, N.M., Glymour, M.M. & Tchetgen Tchetgen, E.J. Practical guidance for conducting mediation analysis with multiple mediators using inverse odds ratio weighting. *Am J Epidemiol* **181**, 349-356 (2015).

6. Hossin, M.Z., Koupil, I. & Falkstedt, D. Early life socioeconomic position and mortality from cardiovascular diseases: an application of causal mediation analysis in the Stockholm Public Health Cohort. *BMJ Open* **9**, e026258 (2019).
